# Vector Saliva in Vaccines for Visceral Leishmaniasis: A Brief Encounter of High Consequence?

**DOI:** 10.3389/fpubh.2014.00099

**Published:** 2014-08-08

**Authors:** Shaden Kamhawi, Hamide Aslan, Jesus G. Valenzuela

**Affiliations:** ^1^Vector Molecular Biology Section, Laboratory of Malaria and Vector Research, National Institute of Allergy and Infectious Diseases, National Institutes of Health, Rockville, MD, USA

**Keywords:** visceral leishmaniasis, sand fly vectors, vector-transmission, salivary proteins as vaccines, Th1 immune response, delayed-type hypersensitivity response

## Abstract

Visceral leishmaniasis (VL) is a vector-borne disease transmitted by phlebotomine sand flies and remains the most serious form of the disease with no available human vaccine. Repeatedly, studies have demonstrated the immunogenicity and protective efficacy of a number of sand fly salivary proteins against cutaneous and visceral leishmaniasis. All *Leishmania* species including agents of VL are co-deposited into the skin together with vector saliva. Generally, the immune response to a protective salivary protein in vaccinated animals is rapid and possibly acts on the parasites soon after delivery into the skin by the bite of an infective sand fly. This is followed by the development of a stronger *Leishmania*-specific immunity in saliva-vaccinated animals compared to controls. Considering that several of the most efficacious protective molecules were identified from a proven vector of VL, we put forward the notion that a combination vaccine that includes a *Leishmania* antigen and a vector salivary protein has the potential to improve vaccine efficacy by targeting the parasite at it most vulnerable stage just after transmission.

## Background

Visceral leishmaniasis (VL), also known as kala-azar, is a systemic vector-borne neglected disease that is fatal if left untreated. There are an estimated 300,000 cases of VL globally with over 20,000 deaths per year, a statistic second only to malaria among parasitic diseases (1). Over 90% of VL cases occur in six countries (Bangladesh, Brazil, Ethiopia, India, South Sudan, and Sudan) where about 300 million people are at risk of infection (1, 2). From 2009 to 2012, an epidemic in South Sudan caused over 28,300 cases and nearly 900 deaths^1^. Other areas have also been affected by recent persistent epidemics of VL in Ethiopia and Kenya^1^.

Visceral leishmaniasis is caused either by *Leishmania donovani* or *L. infantum*. VL caused by *L. donovani* is prevalent in East Africa and the Indian sub-continent and is considered an anthroponosis, while VL caused by *L. infantum* is prevalent in South Europe, North Africa, parts of the Middle East and Latin America (3–6). Phlebotomine sand flies are still considered the primary and stable mode of VL transmission. Different species of sand flies have been incriminated as vectors of VL. *Phlebotomus argentipes* is the only known vector of *L. donovani* in the Indian sub-continent (7–9) and *P. orientalis* represents the main sand fly species transmitting *L. donovani* within countries of East Africa, Saudi Arabia, and Yemen (10, 11). On the other hand, there are several proven vectors of VL in the Eastern Mediterranean among which *P. ariasi* and *P. perniciosus* represent the primary species transmitting *L. infantum* (12, 13), while *Lutzomyia longipalpis* is considered the primary vector of *L. infantum* across Latin America (14, 15).

Despite, its wide distribution and high mortality rate, there are no available human vaccines against VL. Even with recent improvement in treatment (16–19) and the gates initiative for the elimination of VL from the Indian sub-continent^2^, there remains a need to develop a vaccine, particularly when considering the prevalence of infected individuals with subclinical infections that potentially present an uncontrolled source of parasites for the sand fly vector (20). Though the primary function of vector saliva is to facilitate blood feeding (21), a good body of evidence has shown that it modulates host immunity altering the outcome of infection with *Leishmania* and under certain circumstances, protecting from disease (22–24). Here, we give our perspective on the relevance of vector saliva in the transmission of and for vaccines against VL.

## Vector Saliva and Protection from Leishmaniasis

Vaccination with certain immunogenic proteins in saliva of vector sand flies confers protection from leishmaniasis (25–35). Protective molecules have mostly shared a similar property, the induction of a delayed-type hypersensitivity (DTH) response biased toward a Th1 profile. Importantly, animals previously exposed to saliva or vaccinated with a Th1-biased DTH-inducing salivary protein were protected against challenge by infected vector bites (28, 30, 35). This is significant since Peters et al. (36) showed that the innate immune response following sand fly transmission varied significantly from the response induced by needle challenge primarily related to a persistence of a neutrophilic infiltrate at the site of bite enhancing parasite virulence. Additionally, the enhanced virulence of vector-transmission was shown to abrogate protection by *Leishmania* vaccines tested against needle challenge with parasites largely due to the need for a rapid effector immune response (37). Thus, saliva-mediated protection from vector-transmitted leishmaniasis suggests that the immune response to salivary proteins is rapid enough to restrict the establishment of *Leishmania* parasites following vector-challenge. Furthermore, the protection against vector-challenge displayed by animals vaccinated with a defined recombinant salivary protein indicates that the native protein despite its presence among others in saliva of the vector initiated an efficient recall response upon its co-deposition in skin with the parasites (28).

Recently, a study investigating the value of combining a protective salivary vaccine with promising *Leishmania* antigens tested several combinations of PpSP15, a protective salivary protein from *P. papatasi* (31, 33), with live recombinant *L. tarentolae* stably expressing the cysteine proteinases CPA and CPB (38). In both BALB/c and C57BL/6 mice, the animals primed with PpSP15 DNA and boosted with PpSP15 DNA and live recombinant CPA/CPB-expressing *L. tarentolae* exhibited the strongest protection against *L. major* infection followed by the group immunized with both PpSP15 and CPA/CPB-expressing *L. tarentolae* injected in independent sites (38). This study is the first to demonstrate the enhanced protection from leishmaniasis resulting from the inclusion of a vector salivary component to the vaccine.

The significance of vector salivary proteins in *Leishmania* vaccines is made more credible by the observed immunogenicity of saliva in exposed humans (39–41). Gomes et al. (39) first reported on the association between the appearance of antibodies to *L. longipalpis* saliva and the development of a protective cell-mediated immunity to *L. chagasi*. In another study, volunteers experimentally exposed to *L. longipalpis* produced distinct skin reactions at the bite site and displayed an increased frequency of IFN-γ- and IL-10-producing T cells (40). Additionally, the authors demonstrated that PBMC from volunteers maintained an efficient recall response 1 year after their first exposure and produced IFN-γ upon *in vitro* stimulation with saliva that was associated to a significant reduction in macrophage infection rates with *L. chagasi*. More recently, we demonstrated that the DTH response in individuals naturally exposed to bites of *P. duboscqi*, another vector sand fly, persists to mid life (41). Importantly, though PBMC from volunteers showed a Th1, Th2, or a mixed response upon *in vitro* stimulation with saliva, dermal biopsies from bite sites with a DTH response were dominated by macrophages and lymphocytes and exhibited an abundance of IFN-γ indicative of a Th1 milieu (41). Though more studies in humans are needed, the above results demonstrate that repeated exposure to sand fly saliva alters the immune response of humans to the parasites co-deposited into the wound at the site of an infected bite.

## Transmission of Visceral Leishmaniasis and Vector Saliva

Despite reports of vertical transmission of *L. infantum* (42), it is still accepted that VL, caused by *L. donovani* or *L. infantum*, is mostly transmitted by bite of infected phlebotomine sand flies. At the site of bite, the sand fly deposits few parasites (43–45) alongside saliva in the skin. Therefore, though pathology of VL is ultimately the result of failure of internal organs, mainly the spleen and liver, there is a vital phase early after transmission where the few parasites deposited in the skin are at their most vulnerable. We believe it is at this stage that immunity to a salivary protein can potentially exert a profound effect on the survival and ability of the parasites to visceralize. Studies have identified immunogenic salivary proteins from important VL vectors that induce a distinct Th1–DTH response predictive of protection from leishmaniasis (27, 29, 31, 46). In the only study investigating the potential of salivary proteins to protect against VL, LJM19, a Th1–DTH-inducing salivary protein from *L. longipalpis*, a VL vector, conferred powerful protection against progressive VL in vaccinated hamsters (29). LJM19-vaccinated animals displayed a high IFN-γ/TGF-β ratio and inducible NOS expression in the spleen and liver associated to a controlled parasite burden and survival up to 5 months post-infection. In contrast, controls and hamsters vaccinated with other salivary molecules developed progressive fatal VL within the same time frame (29). The long-term systemic protection from *L. chagasi* (*L. infantum*) conferred by immunity to LJM19 was likely driven by the initial immune response to LJM19 in the skin where a distinct DTH response with high expression of IFN-γ was observed 48 h after challenge with uninfected sand flies (29). Due to a shorter course of infection and the ease of assessing disease burden most studies of the protective capacity of immunogenic salivary proteins from saliva of *L. longipalpis* were tested using CL infection models producing promising results. Mice vaccinated with maxadilan, the vasodilator from *L. longipalpis* saliva protected mice against *L. major* infection (34), while vaccination with LJM19, protected hamsters against infections with *L. braziliensis* co-injected with saliva of the natural vector *L. intermedia* (32). LJM11, another Th1–DTH-inducing salivary protein from *L. longipalpis*, conferred partial protection against *L. infantum* in hamsters (29) and a strong protection against infections initiated by needle or vector-challenge with *L. major* in mice (28, 47). Table 1 provides a summary of potential salivary vaccines identified from VL vectors to date.

**Table 1:** **Vaccine candidates identified from saliva of visceral leishmaniasis vectors**.

Sand fly species	Salivary molecule	Immunogenicity	Protection	Animal model	Reference
*L. longipalpis*	Maxadilan	Th1, IgG	*L. major*	Mouse	(31)
*L. longipalpis*	LJM19	Th1/DTH	*L. infantum*, *L. braziliensis*	Hamster	(29, 32)
*L. longipalpis*	LJM11	DTH, IgG	*L. infantum*	Hamster (partial)	(29)
*L. longipalpis*	LJM11	Th1/DTH, IgG2a	*L. major*	Mouse	(28, 47)
*L. longipalpis*	LJM17	Th1/DTH, IgG2a	*L. infantum*	Dog	(27)
*L. longipalpis*	LJL143	Th1/DTH, IgG2a	*L. infantum*	Dog	(27)
*P. ariasi*	ParSP01	DTH		Mouse	(46)
*P. ariasi*	ParSP03	DTH, IgG2a		Mouse	(46)
*P. ariasi*	ParSP25	DTH, IgG1		Mouse	(46)

Studies carried out using CL models of infection have demonstrated that the initial immune response directed against sand fly saliva or one of its proteins gives rise to an accelerated and potent immune response specific to the *Leishmania* parasite (28, 31). The initial saliva-specific immune response is observed as early as 2–6 h after bite up to 1 week post-challenge (29–31, 35). By 2-weeks post-infection, animals vaccinated with a salivary protein mount a stronger *Leishmania*-specific immunity with minimized pathology (28, 31). This supports our hypothesis that the initial immune response to a salivary protein in the skin can potentially alter the nature of the immune response to the parasites long-term and is therefore relevant for protection against both CL and VL.

## Vector Saliva in a Vaccine for Visceral Leishmaniasis

### Rationale

From the above, immunity to a vector salivary protein can potentially have an enormous impact on progression of VL. Visceralizing parasites are initially inoculated into the skin then navigate their way to the viscera in a poorly understood manner. Assuming that for a brief period of time these parasites are in the skin, low in number, and in close proximity to co-inoculated salivary proteins, a vaccine strategy involving immunization with a Th1-inducing salivary protein that would initiate a rapid immune response to itself at the site of bite will adversely impact the vulnerable *Leishmania* parasites while still in the skin. Such a vaccine could potentially enhance the efficacy of a VL vaccine by introducing an additional stage in which the parasites are attacked.

### Diversity of VL foci

The complexity of VL transmission would clearly have an impact on the design and practicality of a salivary vaccine. *L. donovani*, considered an anthroponosis, is transmitted by only one species of sand flies in the Indian sub-continent but has multiple vectors in East Africa (7–11, 48). A similar situation exists for zoonotic VL due to *L. infantum* where across Latin America transmission is mostly by a single primary vector while along the Eastern Mediterranean, over six species of sand flies have been incriminated as major VL vectors (12–15). Foci where transmission involves multiple vectors would be more challenging compared to those where a vaccine needs to target a single vector species. Under these conditions, the future for salivary antigens is most likely in vaccines tailored for specific regions. Nonetheless, in several of the most important foci of VL including India, Sudan, and Latin America there is but one primary vector sand fly species, *P. argentipes*, *P. orientalis*, and *L. longipalpis*, respectively (8–10, 14, 15, 49), a situation where a tailored vaccine may be justified.

### Challenges and solutions

As mentioned above, in foci with a primary vector, inclusion of a salivary protein in a leishmaniasis vaccine can potentially enhance its efficacy. On the other hand, certain VL foci such as those in the Eastern Mediterranean region have multiple incriminated VL vectors (12, 48). For such foci, a salivary molecule with the appropriate immunogenicity needs to have close homologs in most sympatric vector species, creating a considerable obstacle. We are now addressing whether priming with a salivary protein and boosting with both the salivary antigen and a *Leishmania* antigen will drive a *Leishmania*-specific immunity strong enough to overcome the obstacle presented by specificity of vector salivary molecules. If successful, incorporating the best of the immunogenic salivary proteins with the most promising *Leishmania* antigens may present an opportunity for a pan leishmaniasis vaccine. Here, we must underscore that though a robust immunity to *Leishmania* driven by a preceding immunity to saliva has been demonstrated (28, 31), it was always generated by a challenge with virulent live parasites. It remains to be validated whether a similar level of protective immunity can be achieved with a single antigen. Considering the payback, it is a question worthy of further exploration.

### Further considerations

Identifying salivary molecules from VL vectors that can induce a Th1-biased immunity in humans should be prioritized. Expression libraries of the secreted salivary proteins of several VL vectors are available (46, 50–53) and high throughput expression of endotoxin-free recombinant proteins of high purity has been achieved (28, 54). Developing a rapid screening assay using PBMC of healthy exposed volunteers stimulated with recombinant salivary proteins from VL vectors could rapidly reveal immunogenic antigens appropriate for further exploration as protective vaccine candidates using animal models. Additionally, we recently developed a hamster model of vector-transmitted progressive VL (55) that can further facilitate the prioritization of salivary vaccine candidates found immunogenic in humans. Here, it is important to emphasize the need to begin the search for a vaccine candidate using human cells (56). Multiple leishmaniasis vaccine candidates protected various animal models but failed to protect humans (57). This is not surprising considering that the initiation of a Th1 cellular immunity such as that induced by salivary molecules and required for protection against leishmaniasis implies efficient recognition of specific antigenic epitopes by human leukocyte antigen I (HLA-I) and HLA-II molecules for presentation to T cells (58). However, unlike anthroponotic VL where humans are the only vaccine target, zoonotic VL needs to target dogs as the domestic reservoirs and the primary source of infection to sand flies and humans (12, 48, 59, 60). Therefore, in addition to humans, salivary molecules immunogenic in dogs such as those reported for *L. longipalpis* (27), should also be considered for a canine vaccine.

## Shades of Gray

Though, we tend to put *Leishmania* species in clear-cut categories, nature tells us otherwise. The unique polymorphic nature of leishmaniasis and the plasticity of *Leishmania* parasites continue to confound efforts toward disease control. There are several reports where a single parasite strain commonly causing dermatotropic symptoms manifests as a visceral infection and vice versa (61–63). Specifically, we still do not understand why *L. infantum*, associated mainly with VL, causes only cutaneous disease in some regions (64). Similarly, *L. donovani* zymodeme MON-37, the parasite strain previously associated exclusively with VL in India and East Africa, has been identified as the causative agent in recently established foci of CL in Sri Lanka (65, 66). These unusual manifestations of leishmaniasis clearly demonstrate how little we understand the factors contributing to disease. The fact that dermotropic *L. infantum* genotypes can disseminate and cause severe VL in immunosuppressed individuals is indicative of the importance of host susceptibility in the outcome of infection with *Leishmania* parasites (67). But is the etiology of leishmaniasis mainly due to host immunity or are environmental pressures, vector-derived factors and evolution of the parasite itself equally significant? Most likely the form of leishmaniasis contracted is the consequence of all the aforementioned factors. Hence, we need to keep an open mind in our search for vaccines and perhaps entertain the option of a tailored vaccine enhanced by a salivary component of a primary vector in foci of high morbidity and mortality.

## Conclusion

To date, a human vaccine against any form of leishmaniasis is non-existent. There is strong evidence that certain proteins in sand fly vector saliva can: (1) induce a Th1–DTH immune response; (2) protect against both CL and VL; (3) protect against vector-initiated CL; and (4) induce a *Leishmania*-specific robust immunity after challenge with minimized pathology. Considering the above, should not salivary proteins of vector sand flies be given serious consideration as candidate components in a *Leishmania* vaccine?

## Conflict of Interest Statement

The authors declare that the research was conducted in the absence of any commercial or financial relationships that could be construed as a potential conflict of interest.

## Footnotes

^1^www.who.int/leishmaniasis

^2^www.gatesfoundation.org
